# Quorum Sensing and Toxin Production in *Staphylococcus aureus* Osteomyelitis: Pathogenesis and Paradox

**DOI:** 10.3390/toxins12080516

**Published:** 2020-08-12

**Authors:** Casey E. Butrico, James E. Cassat

**Affiliations:** 1 Department of Pathology, Microbiology, and Immunology, Vanderbilt University Medical Center, Nashville, TN 37232, USA; casey.e.butrico@vanderbilt.edu; 2 Department of Pediatrics, Division of Pediatric Infectious Diseases, Vanderbilt University Medical Center, Nashville, TN 37232, USA; 3 Vanderbilt Center for Bone Biology, Vanderbilt University Medical Center, Nashville, TN 37232, USA; 4 Department of Biomedical Engineering, Vanderbilt University, Nashville, TN 37232, USA; 5 Vanderbilt Institute for Infection, Immunology, and Inflammation (VI4), Vanderbilt University Medical Center, Nashville, TN 37232, USA

**Keywords:** osteomyelitis, *Staphylococcus aureus*, toxin, accessory gene regulator, quorum, virulence, infectious, pathogenesis, bone

## Abstract

*Staphylococcus aureus* is a Gram-positive pathogen capable of infecting nearly every vertebrate organ. Among these tissues, invasive infection of bone (osteomyelitis) is particularly common and induces high morbidity. Treatment of osteomyelitis is notoriously difficult and often requires debridement of diseased bone in conjunction with prolonged antibiotic treatment to resolve infection. During osteomyelitis, *S. aureus* forms characteristic multicellular microcolonies in distinct niches within bone. Virulence and metabolic responses within these multicellular microcolonies are coordinated, in part, by quorum sensing via the accessory gene regulator (*agr*) locus, which allows staphylococcal populations to produce toxins and adapt in response to bacterial density. During osteomyelitis, the Agr system significantly contributes to dysregulation of skeletal homeostasis and disease severity but may also paradoxically inhibit persistence in the host. Moreover, the Agr system is subject to complex crosstalk with other *S. aureus* regulatory systems, including SaeRS and SrrAB, which can significantly impact the progression of osteomyelitis. The objective of this review is to highlight Agr regulation, its implications on toxin production, factors that affect Agr activation, and the potential paradoxical influences of Agr regulation on disease progression during osteomyelitis.

## 1. Introduction

Osteomyelitis, or inflammation of bone, most frequently occurs as a result of bacterial infection. *Staphylococcus aureus* is the most commonly isolated bacterium from infectious osteomyelitis and can result in acute or chronic disease [1]. While osteomyelitis infections most frequently manifest in children from hematogenous spread of organisms colonizing the skin or nares, contiguous spread from trauma or surgery is more common in adults [1,2]. The ability of *S. aureus* to spread to bone from other tissues is in part dependent upon the ability to express toxins that mediate dissemination [3]. The study of human disease and the development of animal models are both important for understanding the pathogenesis of *S. aureus* osteomyelitis [4,5,6,7]. In bone, *S. aureus* can reside in a number of different microenvironments including necrotic bone fragments (known as sequestra), within bone marrow abscesses, under the periosteum of intact bone, or within the canalicular system of cortical bone [1,8,9]. Regardless of the precise niche within skeletal tissues, *S. aureus* often exists in multicellular structures, known as microcolonies. Within these microcolonies, *S. aureus* is able to adapt and respond to resource fluctuations and bacterial density.

The capability of *S. aureus* to grow in a multitude of host tissues, including bone, suggests remarkable flexibility in responding to heterogeneous host immune responses and local nutrient availability. *S. aureus* responds to immune and nutrient stress in part by secreting virulence factors, including enzymes that degrade host tissues, cytolytic toxins that target host cells, and molecules that incapacitate immune defenses [10]. For the purposes of this review, toxins are broadly characterized as proteins produced by *S. aureus* and secreted into the extracellular matrix or presented on the bacterial surface that have detrimental effects on host cellular function or physiology. Importantly, the production of bacterial toxins has less favorable tradeoffs, including an increased energy requirement and the potential to incite host immune defenses and increase inflammation [11,12]. Accordingly, *S. aureus* senses a combination of bacterial- and host-derived environmental cues to regulate the production of toxins that are essential for full *S. aureus* pathogenicity during osteomyelitis [13]. The accessory gene regulator (Agr) system is a key mediator of bacterial responses to environmental stimuli and regulates the production of many *S. aureus* factors to promote bacterial survival and dissemination [3]. This review serves to summarize the recent and historic findings regarding the regulation and function of *S. aureus* toxins, with a particular emphasis on those influenced by quorum sensing via Agr during osteomyelitis.

## 2. Conserved Mechanisms of Two-Component Systems Apply to Quorum-Mediated Virulence Regulation in *S. aureus*

Gram-positive and Gram-negative bacteria have the ability to sense and respond to the environment through two-component regulatory systems, which classically consist of a histidine kinase and a response regulator [14]. Depending on the two-component system, response regulators can be activated in their de-phosphorylated and/or phosphorylated state in response to stimuli recognized by the histidine kinase. Response regulators typically function as transcription factors that bind to bacterial DNA to alter transcriptional responses to intra- or extracellular stimuli [13,15]. Interestingly, some activated response regulators can cross-regulate transcription of genes associated with other two-component systems [16,17].

Many bacterial species, including *S. aureus*, possess specialized two-component systems to survey and respond to the density of genetically similar cells. This process, known as quorum sensing, allows *S. aureus* to detect the cellular density in a given niche via production of an autoinducing peptide (AIP). *S. aureus* AIP activates the Agr two-component system when it reaches a threshold concentration [18,19]. The Agr two-component system is responsible for controlling critical toxin genes involved in *S. aureus* pathogenesis, including hemolysins and phenol-soluble modulins (PSMs), while also repressing production of proteins involved in bacterial adherence and aggregation, such as coagulase (Coa) and von Willebrand factor-binding protein (vWbp) [7,13,20]. The complexity of *S. aureus* toxin production is compounded by the effects of the Agr quorum system, including toxin activation and decreased adherence, which might be counterproductive for *S. aureus* pathogenicity during chronic infection. For example, mutations within the *agr* locus result in repression of bacterial toxin production that can lead to evasion of immune recognition during persistent *S. aureus* infection [18,21].

Although the Agr system strongly contributes to staphylococcal virulence, other two-component systems and regulatory proteins, including staphylococcal accessory regulator A (SarA), catabolite control protein A (CcpA), *S. aureus* exoprotein expression (SaeRS), and staphylococcal respiratory response AB (SrrAB) also influence virulence factor regulation. Importantly, these two-component systems can regulate virulence factors in both Agr-dependent and Agr-independent manners [22,23,24,25]. While Agr only responds to AIP concentration, multiple environmental cues, including oxygen concentration, oxidative stress, and nutrient availability, influence *S. aureus* toxin production. Therefore, the local tissue environment of the infection alters *S. aureus* toxin production and is controlled at multiple levels through the Agr system.

Bone has relatively lower physioxia than many tissues and becomes increasingly hypoxic over the course of infection [26]. Oxygen availability is further restricted through the formation of *S. aureus* abscesses and biofilms. Reduced oxygen tension promotes *S. aureus* cytotoxin production in a SrrAB-dependent and -independent manner, thereby mediating pathogenesis [26]. In addition to the ability to sense and respond to changes in oxygenation, *S. aureus* can also adapt based on amino acid and metabolite availability with the regulatory proteins CodY and CcpA. SrrAB, CodY, and CcpA all influence transcription from *agr* locus promoters, albeit through different mechanisms [27,28]. Finally, SaeRS responds to stress induced by neutrophils and other host immune cells to influence the Agr network of virulence genes [7]. Thus, multiple two-component systems and regulatory proteins facilitate *S. aureus* survival in bone and are intricately linked to Agr quorum sensing. These mechanisms are discussed in more detail below.

## 3. Agr Signaling Mechanisms Regulate Toxin and Immunomodulatory Protein Production during *S. aureus* Osteomyelitis

A number of staphylococcal virulence factors that are directly or indirectly regulated by the Agr system contribute to the pathogenesis of osteomyelitis, including cytolytic and immunomodulatory proteins. In the sections that follow, we highlight the regulatory architecture of the Agr system, the mechanisms of regulation of toxins related to osteomyelitis, and the impact of other regulators and two-component systems on Agr and Agr-regulated targets.

### 3.1. Agr Signaling Components Interact to Control Virulence Genes

**Figure 1:**
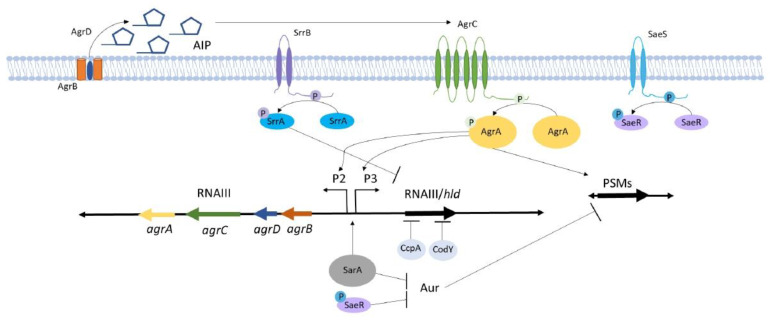
Accessory gene regulator (Agr) signals through self-regulation and crosstalk with other two-component systems. Quorum-mediated Agr signaling is influenced by regulatory proteins, staphylococcal accessory regulator A (SarA), carbon catabolite control protein A (CcpA), and CodY and two-component systems, staphylococcal respiratory response AB (SrrAB) and *Staphylococcus aureus* exoprotein expression response regulator (SaeRS), which are responsive to oxygen (SrrAB) and nutrient availability (SaeRS), as well as host immune stress. AgrB facilitates the formation of extracellular AIP from the AgrD precursor. Agr signaling occurs through AIP-mediated signal transduction at a threshold concentration that activates AgrC phosphotransferase activity to phosphorylate AgrA. AgrA activates the transcription of RNAIII via P3 to produce virulence factors implicated in *S. aureus* acute osteomyelitis. AgrA binding to P2 results in transcription of RNAII, leading to additional *agrA*, *agrC*, *agrD*, and *agrB* mRNA. Phosphorylated AgrA also enhances the transcription of phenol-soluble modulins (PSMs) directly. SarA and activated SaeR indirectly augment PSM production via inhibition of the protease Aureolysin (Aur).

The components of the Agr system are co-regulated through the cross-activation of divergent promoters, P2 and P3, which generate RNAII and RNAIII, respectively (Figure 1). RNAII contains the sequences for the translation of AgrD, the precursor for the extracellular quorum peptide, AIP, and its maturation and export protein, AgrB. RNAIII also encodes AgrC, the sensor histidine kinase of the two-component system and its cognate response regulator, AgrA. Activation of the P2 promoter occurs in response to AgrC phosphorylation of AgrA when extracellular AIP is detected [29,30]. Therefore, a positive feedback exists for the translation of AgrC and AgrA to receive and transduce the quorum-dependent signal. Phosphorylated AgrA has a high affinity for the P2 promoter, which facilitates the continued transcription of *agrA-D* to produce extracellular AIP. AgrA also binds to the P3 promoter to initiate transcription of RNAIII, although the affinity for this promoter is lower, allowing for AIP production prior to RNAIII transcription [30,31]. Downstream transcriptional and translational regulation of toxin-related genes is mediated, in part, by the RNAIII transcript [32].

Phosphorylation of AgrA directly regulates several genes coding for toxins (e.g., PSMs) and indirectly represses genes involved in cell wall surface attachment and biofilm formation (see below) [33,34,35]. Additionally, Agr virulence factor regulation occurs at the transcriptional and translational levels via binding of the regulatory RNAIII to virulence-related transcripts [35]. The RNAIII regulatory RNA has a number of highly conserved domains between staphylococcal species that are likely functional domains for these regulatory processes [36]. Surface-associated molecules that function in bacterial aggregation, including coagulase (Coa), are negatively regulated at the transcriptional level via RNAIII [20,37]. RNAIII controls the transcription of toxin and immunomodulatory genes including alpha-hemolysin (*hla*), beta-hemolysin (*hlb*), toxic shock syndrome toxin-1 (*tst*), and staphylococcal serine protease (*sspA*) by binding within the promoter regions of these genes [13,38,39]. While RNAIII increases the transcription of *hla*, it also promotes translation of the trans-encoded *hla* mRNA by binding to the untranslated region to resolve an intramolecular base pairing that typically blocks the ribosomal binding site [33].

In addition to regulating toxin production, RNAIII also controls immune evasion proteins, the second immunoglobulin-binding protein (Sbi), MHC class II analog protein (Map), and protein A (SpA). RNAIII represses translation of *sbi* mRNA through direct interaction with the translation initiation sequence [40]. Contrary to the effects of RNAIII base pairing with *sbi*, RNAIII increases Map production, likely through RNAIII-promoted translation initiation, although the precise mechanism is unknown [41]. RNAIII represses transcription as well as translation of SpA, but it also exerts a third level of regulatory control by promoting the degradation of *spa* mRNA via double-strand specific endoribonuclease activity. The mechanisms of SpA translational inhibition and arrest are both initiated as the 3′ end domain of RNAIII binds to the complementary 5′ domain of *spa* mRNA. This results in a loop–loop interaction through annealing and prevents in vitro formation of the translation initiation complex. Simultaneously, the loop–loop interaction also recruits RNase III to irreversibly arrest translation [42].

RNAIII further regulates the production of transcription factors that subsequently control toxin and adherence genes. For example, RNAIII stabilizes and increases the production of *mgrA*, which encodes a global transcription regulator and, in turn, controls over 350 genes involved in virulence, antibiotic resistance, autolysis, and biofilm formation [43]. RNAIII also acts as an anti-repressor by binding the mRNA of the repressor of toxins (Rot) protein at the ribosome-binding site. Rot typically inhibits translation of many toxins and surface proteins and positively regulates multiple virulence factors implicated in osteomyelitis, including SpA and SarA family proteins [44,45].

Finally, while RNAIII is the primary effector involved in virulence regulation, activated AgrA can also directly influence the toxin gene expression. In particular, the transcription of PSMs are upregulated by AgrA binding to the promoter region, and Agr-mediated PSM production contributes to bone loss during osteomyelitis [35]. Thus, the Agr regulatory system can control the production of toxins and other virulence factors through direct gene activation/repression by AgrA or RNAIII, translational control through RNAIII base pairing with mRNA, and via RNAIII mRNA duplex formation that promotes endonuclease activity for mRNA degradation. Importantly, while Agr promotes toxin production, it also inhibits factors associated with bacterial persistence [21,46]. Agr-mediated regulation of bacterial toxins and factors associated with persistence has been implicated in the pathogenesis of *S. aureus* osteomyelitis in a number of studies (Table 1). The following sections will highlight Agr-regulated toxins implicated in osteomyelitis.

### 3.2. S. aureus Cytolytic Toxins Regulated by Agr

#### 3.2.1. Phenol-Soluble Modulins (PSMs)

PSMs are key virulence factors produced by *S. aureus* that have cytotoxic effects on many cell types, including bone cells, and contribute to the pathogenesis of osteomyelitis. The genes encoding PSMs are located in three distinct chromosomal locations, which encode PSMα, PSMβ, and δ-toxin (Hld). *Hld* is co-transcribed from the same promoter as RNAIII, while *psmα* and *psmβ* are produced through AgrA-mediated transcription [53]. At high concentrations, these small peptides destabilize lipid bilayers due to their amphipathic nature, thereby dysregulating ion flux and initiating host cell lysis when exported through the phenol-soluble modulin transporter (Pmt) [54]. While PSMs are able to induce cytotoxicity in host cells through receptor-independent pore formation, they also activate neutrophils through formyl-peptide receptor 2 (FRP2) to induce proinflammatory cytokine production [55,56,57]. This is particularly relevant to osteomyelitis, as proinflammatory cytokines increase bone degradation via enhanced production and activity of bone resorbing cells, known as osteoclasts [58]. PSM production from phagocytosed, intracellular *S. aureus* is necessary and sufficient to induce neutrophil death [57]. Intracellular production of *S. aureus* PSMα 1–4 also facilitates bacterial escape from osteoblasts, which could promote dissemination during osteomyelitis [7,55]. *S. aureus* α-type PSMs have deleterious effects on osteoblasts that result in dramatic changes in bone remodeling in addition to promoting the intracellular survival of the bacteria [7,55]. Furthermore, our laboratory has demonstrated the role of SrrAB in the regulation of PSMs during oxygen-limited growth, as well as the role of the Sae-regulated protease aureolysin (Aur) in degrading these peptides [7,26]. At this point, it is unclear whether β-type PSMs contribute to osteomyelitis. Nevertheless, many community-acquired strains of *S. aureus*, including those frequently isolated from patients suffering from osteomyelitis, express PSMs at a high level and are consequently highly cytotoxic.

#### 3.2.2. Alpha-Hemolysin (Hla)

Hla is a polypeptide translated by *S. aureus* as a soluble monomer, which assembles upon contact with the host lipid bilayer [59]. Hla oligomers are produced in a RNAIII-dependent manner. RNAIII inhibits formation of a stem–loop structure within the *hla* mRNA, as described above [34]. When a central glycine-rich loop becomes occluded from the hexameric structure, Hla forms oligomers that assemble to form a pore and induce host cell cytotoxicity [60,61]. In order to bind to host cell membranes and oligomerize, Hla engages the disintegrin and metalloproteinase domain-containing protein 10 (ADAM10) receptor [62,63]. ADAM10 is responsible for proteolytically processing a number of biologically active signaling molecules and physiological functions in host cells [63]. Thus, targeting ADAM10 remains a therapeutic strategy of continued development to prevent host cell cytotoxicity during *S. aureus* infection [11].

Exposure of monocytic and epithelial cells to Hla results in nuclear factor kappa B (NF-κB) and nucleotide-binding domain leucine-rich repeat and pyrin domain containing receptor 3 (NLRP3) inflammasome activation and induction of interleukin 1 (IL-1) and 18 (IL-18) [64,65]. Based on these in vitro studies, we hypothesize that the production of these pro-inflammatory cytokines may contribute to tissue damage in bone and muscle tissue, although further studies are necessary to confirm. Additionally, during septic *S. aureus* infections, there is a Hla-dependent systemic increase in interleukin 6 (IL-6), a known contributor to osteoclast activation. IL-6 may therefore result in greater amounts of degradative bone resorption due to the inflammatory environment induced by Hla [66]. Within the musculoskeletal system, Hla has been implicated as a potent chondrocyte toxin and causes rapid death of these cells within cartilage. Chondrocyte cytotoxicity is a particular concern during septic arthritis, where it significantly impacts joint destruction [67].

#### 3.2.3. Panton–Valentine Leukocidin (PVL)

Another *S. aureus* toxin with tissue-specific implications during infection is PVL, a virulence factor strongly associated with community-acquired MRSA [68]. PVL is composed of a S component, encoded by *lukS*, and a F component, encoded by *lukF*. Once translated, these polypeptides form pores within host cell membranes by oligomerizing [69]. The stem domains of both subunits insert into the membrane, forming a beta barrel pore and resulting in osmotic lysis [70]. Using this pore-forming mechanism, PVL induces neutrophil apoptosis or necrosis, depending on the concentration of toxin, preventing bactericidal killing by these innate immune cells [71]. PVL also binds to monocytes and macrophages and activates the NLRP3 inflammasome, which leads to production of proinflammatory cytokines [72].

PVL expression correlates with the prevalence, duration, and severity of osteomyelitis, as well as the extent of inflammation in clinical isolates [73,74,75]. Additionally, clinical studies link PVL-positive *S. aureus* bone and joint infections with severe sepsis and muscular tissue inflammation in pediatric patients [74,76]. In a rabbit model of osteomyelitis, PVL facilitated bacterial spread to musculoskeletal tissues [71]. In contrast, models of *S. aureus* skin and lung infection do not exhibit significant increases in pathogenesis with PVL-positive strains in BALB/c mice [77]. Species-specific effects of PVL on *S. aureus* infection severity result from S component binding to C5aR coupled with F component CD45 receptor-specificity. However, the PVL F component binds murine CD45 with 10-fold lower affinity [78,79]. Thus, based on the limited number of studies with humanized animal models, we hypothesize that PVL expression may increase *S. aureus* osteomyelitis disease severity and dissemination in human clinical disease, and this phenotype is partially recapitulated in rabbit models. However, the species-specific receptor of PVL reveals a limitation in animal models of *S. aureus* infection and complicates virulence studies.

### 3.3. S. aureus Immunomodulatory Molecules Regulated by Agr

#### 3.3.1. Protein A (SpA)

SpA is an immunoglobulin (Ig)-binding protein that is both secreted into the extracellular environment and presented on the surface of *S. aureus* [36]. RNAIII represses SpA through a number of mechanisms, as described above [40]. The immunomodulatory functions of SpA affect both innate and adaptive responses to invasive *S. aureus*. SpA binds the Fc region of antibodies, thereby inhibiting Fc-mediated effector functions [80]. However, SpA also binds B cell receptors, thereby inducing B cell death and preventing the production of *S. aureus* specific antibodies [81,82]. The superantigen effects of SpA against B cells significantly alter the breadth of the humoral immune response by disrupting the proliferation of plasmablasts, promoting short-lived B cells, and decreasing titers of antigen-specific antibodies [83]. In addition to these well-characterized immunomodulatory functions, SpA may have direct effects on skeletal homeostasis in the context of *S. aureus* infection. SpA binds directly to bone forming osteoblasts via Tumor Necrosis Factor Receptor 1 (TNFR-1) to prevent proliferation, induce apoptosis, and inhibit the mineralization capacity of osteoblasts in vitro [75,84]. Binding of TNFR-1 also triggers activation of NF-κB, which leads to an increase in the osteoclastogenic cytokine IL-6 [85]. Osteoblast receptor activator of nuclear factor kappa-B ligand (RANKL) production increases in a partially SpA-dependent manner, thereby stimulating osteoclastogenesis and subsequent bone resorption [84,86]. Another mechanism by which SpA may augment osteoclastogenesis is through increasing the rate of pre-osteoclast migration toward RANKL expressing osteoblasts [86]. Finally, SpA directly induces osteoclastogenesis by binding TNFR-1 and Epidermal Growth Factor Receptor (EGFR) on osteoclast precursor cells [87]. In summary, SpA has numerous immunomodulatory roles that dampen innate and adaptive immune responses while increasing inflammatory bone resorption during *S. aureus* osteomyelitis.

#### 3.3.2. MHC Class II Analog Protein (Map)

Map, also known as extracellular adherence protein (Eap), is a *S. aureus* immunomodulatory protein with a secondary function in promoting bacterial adhesion. Map translation is mediated through a similar Agr-dependent mechanism as Hla, whereby RNAIII binding to *map* mRNA alters protein production [34,41]. However, rather than inhibiting translation, RNAIII anneals to *map* and acts as an antisense RNA to form an RNA duplex, thereby facilitating translation initiation [41]. Once translated, Map interacts with intracellular adhesion molecule 1 (ICAM-1) to mediate adhesion to endothelial cells [88]. However, this molecule also blocks the lymphocyte function-associated (LFA-1) antigen on the surface of neutrophils and ICAM-1 on endothelial cells to prevent leukocyte adhesion and extravasation to the infection site [88,89]. Additionally, in a mouse model of osteomyelitis infection, Map alters T cell function to promote *S. aureus* persistence during infection [90]. In particular, Map attenuates protective cellular immunity by decreasing the rates of T cell proliferation and reducing delayed-type hypersensitivity in response to an antigen challenge [90].

### 3.4. S. aureus Coagulases Regulated by Agr

#### 3.4.1. Staphylocoagulase (Coa)

*S. aureus* is capable of producing three coagulases, clumping factor A (ClfA), Coa, and vWbp, although only the latter two are regulated directly through Agr [91,92,93]. Coagulases are enzymes that bind to and activate prothrombin to convert fibrinogen to fibrin and promote blood clotting. *S. aureus* Coa is implicated in osteomyelitis and is repressed by the Agr system via RNAIII transcription inhibition [20,37,94,95]. This process is necessary for *S. aureus* to colonize host tissue and influences pseudocapsule formation around the staphylococcal abscess community for bacterial containment and defense against invading innate immune cells. In bone, *S. aureus* Coa inhibits proliferation and induces apoptosis of osteoblasts [75]. Coa leads to decreased bone formation, increased RANKL expression, and ultimately, increased bone resorption via stimulation of osteoclasts [75]. Interestingly, antibody-mediated neutralization of this antigen confers protection against future challenge with *S. aureus* in a bacteremia model of infection, suggesting that targeting this molecule during invasive infection could lead to beneficial clinical effects [96]. Additional studies are necessary to determine if these findings can be extrapolated to osteomyelitis.

#### 3.4.2. Von Willebrand Factor-Binding Protein (vWbp)

Similarly to Coa, *S. aureus* uses vWbp to assemble a fibrinogen/fibrin microcolony-associated meshwork (MAM) during growth in host tissues [97]. This protective meshwork assists in bacterial evasion of phagocytosis by innate immune cells by forming a dense fibrin structure beyond the pseudocapsule. Despite their structural similarities, vWbp functions through a mechanism that differs from Coa. Coa binds primarily to soluble fibrinogen, while vWbp does not demonstrate binding preference between soluble and surface-bound fibrinogen, resulting in a lack of competition for binding [98]. While Agr quorum sensing is necessary for late-stage microcolony dispersal, vWbp facilitates formation of a microcolony-associated meshwork for bacterial persistence [97]. The role of vWbp in osteomyelitis is unknown, and future research is necessary to determine the extent of coagulase involvement in disease pathogenesis.

## 4. Regulatory Control of AIP Is Strain and Species Specific

Different strains of *S. aureus* produce distinct AIP molecules that can either activate or inhibit the Agr system in other staphylococcal strains and species. The *agr* sequence homology between *Staphylococcus* species is less than ten percent amino acid sequence conservation, but the resulting lineages have remained stable through generations [99]. As such, *S. aureus* strains are classified into one of four groups based on their cross-activation or -inhibition of the Agr pathway in response to AIP from other strains [100]. Within these clonal groups, *agrD* and *agrB* have distinct nucleotide and amino acid polymorphisms that confer specificity for processing of receptor-ligand interactions [100,101]. In fact, in addition to *agrD* and *agrB* co-evolving within *S. aureus* groups, distinct segments are responsible for AgrB processing of AgrD to AIP [102]. AgrC also evolved with each staphylococcal strain to receive the proper AIP signal within the cytoplasmic membrane, while the signal transducing phosphorylation site is highly conserved [13,100,101]. In vitro, activation of Agr occurs via AIP exposure from a *S. aureus* strain within the same group, while members of other groups may be inhibited by this exposure [101]. Therefore, bacterial interference in staphylococci is mediated, in part, by secreted AIP molecules that suppress virulence factor production in strains or species of different agr types. This unique form of interstrain virulence regulation has the ability to incite competition between strains without the need to produce unique virulence factors. The *agr* type competition extends to commensal coagulase-negative staphylococci species including *Staphylococcus epidermidis* [103]. In fact, commensal species with distinct *agr* types have the ability to attenuate virulence in pathogenic species when co-colonizing mucosal surfaces, thereby protecting the host from *S. aureus* virulence factors regulated by Agr [103,104]. Polymicrobial *S. aureus* infections are frequently characterized in post-traumatic osteomyelitis and are associated with more severe patient outcomes [105]. In other disease models, co-infecting bacterial species, including *Pseudomonas aeruginosa*, augment *S. aureus agr* expression [106]. Cross-kingdom Agr interactions were also identified in polymicrobial infections with *S. aureus* and fungal species, *Candida albicans* [107]. However, further studies are necessary to elucidate the polymicrobial interactions that occur specifically during osteomyelitis and to determine how these interactions impact disease pathogenesis.

## 5. *S. aureus* Clinical Isolates with *agr* Mutations

### 5.1. Characteristic S. aureus agr Mutations Are Isolated from Patients with Chronic Infection and Have the Ability to Revert to Wildtype

*Agr* mutants are frequently isolated from human infections [108,109]. This is important to consider, as Agr-negative strains may have a particular fitness advantage in chronic diseases and are associated with increased mortality in patients with bacteremia [110]. While Agr-negative strains are generally associated with persistent infection, multiple genetic changes in the *agr* locus can increase virulence during sepsis [111]. In 2019, Morikawa and colleagues discovered that a fraction of Agr-negative *S. aureus* mutants revert their Agr activity to wildtype when serially passaged [112]. This finding suggests that *S. aureus* is capable of obtaining the full fitness advantage of Agr-positive virulence factor production, while also benefiting from an Agr-negative chronic infectious state. The *agr* locus is genetically unstable, and two particular *agr* mutations are associated with mutation and reversion: (1) a genetic duplication plus inversion and (2) a 3′ poly(A) tract genetic alteration [112]. Slipped-strand mispairing frequently occurs in short sequence repeat regions such as the poly(A) tract and is a form of phase variation within other virulence factor genes [113,114]. Mutations in the 3′ region of *agrA* have been identified both in clinical isolates where the mutations are generated during the course of infection as well as in non-hemolytic laboratory strains, including RN4220. Poly(A) tract mutations in the *agrA* gene result in delayed RNAIII production and failure to translate hemolysins [114]. Mutations that decrease *agrA* production likely have effects on *S. aureus* response to non-quorum mediated environmental signals due to the complex and intertwined regulation of other two-component systems. In contrast, frameshift insertions and deletions and non-synonymous single nucleotide polymorphisms within *agrA* and *agrC* hotspots are more frequent mutations that permanently inhibit *S. aureus* from utilizing the Agr regulatory system [114]. The instability of the *agr* locus promotes mutation but allows for reversion of certain *agr* mutations back to wildtype.

### 5.2. Agr Expression Is Temporally Regulated to Promote S. aureus Adhesion and Toxin Production at Distinct Phases of Infection

*S. aureus* abscess formation within host tissues, including bone, occurs in a number of phases that expose the bacteria to altered nutrient concentrations and host-derived defenses [115]. As a result, autoinduction of Agr classically occurs in vitro during late and stationary phases of *S. aureus* growth, at a density that exceeds the AIP quorum sensing threshold [93]. In the face of immune cell insults and within phagocytic compartments, Agr coordinates the production of toxins and immunomodulatory factors. Phagocytosis is thought to induce Agr signaling, in part due to the physical containment of *S. aureus* that allows for the accumulation of AIP within phagosomes [116]. Importantly, Agr-negative planktonic *S. aureus* strains can revert to Agr-positive strains within phagosomes of neutrophils [112]. This suggests the bacteria may use Agr phase variation as a cryptic strategy for continued infection while retaining the ability survive phagocytosis. A tradeoff for the expression of toxin genes is the fitness cost of *S. aureus* to produce these factors [112]. Due to the energetic costs of expressing Agr-regulated toxins and the inhibitory effects of Agr on adhesion protein production, chronic phases of infection may favor functional Agr inactivation.

### 5.3. The Agr System Has Paradoxical Effects during Infection

**Figure 2:**
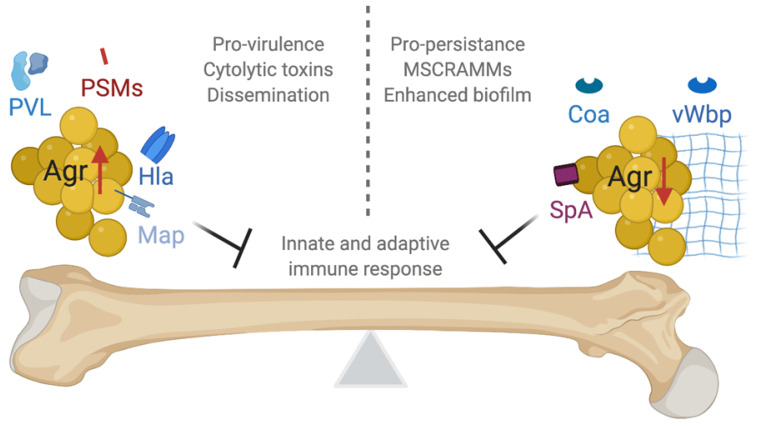
Paradoxical benefits of *agr* expression and inhibition/mutation on *S. aureus* fitness during osteomyelitis. *S. aureus* strains produce virulence factors including cytotoxic proteins, such as phenol-soluble modulins (PSMs) and Panton–Valentine leucocidin (PVL) that insert into the membranes of neutrophils and bone cells to induce cell death in an Agr-dependent manner. *S. aureus* strains with high *agr* expression also produce alpha-toxin (Hla), which induces pro-inflammatory cytokine production and enhances bone resorption. Additionally, the production of MHC class II analog protein (Map) results in suppression of T cell immunity. *S. aureus* strains with mutations in *agr* are commonly isolated from chronic infections, whereby the bacteria exhibit characteristics that enable persistent colonization. Coagulase (Coa) and von Willebrand factor-binding protein (vWbp) contribute to formation of fibrin matrices that facilitate bacterial attachment. Staphylococcal protein A (SpA) increases in abundance in the absence of RNAIII as well and has a number of immunomodulatory functions.

Agr controls expression of toxins that increase *S. aureus* pathogenicity and disease severity; however, inactivating *agr* mutations often occur in *S. aureus* clinical isolates (Figure 2). While it is clear that *S. aureus* Agr positively regulates many virulence factors, including toxins, that allow for bacterial survival in the face of host immune cell defenses, activation of Agr also decreases the presence of factors associated with chronic infection. Agr activation results in repression of many microbial surface components recognizing adhesive matrix molecules (MSCRAMMs) involved in host tissue modification that facilitate bacterial adherence. Agr also regulates production of proteases that degrade adhesion proteins including MSCRAMMs, coagulases, and cytolytic factors such as PSMs [7,91,117]. On the other hand, Agr-negative strains exhibit a fitness advantage under antibiotic stress and have been associated with greater rates of mortality and duration of bacteremia [110,118,119]. In 2019, Gor et al. revealed that the evolutionary tradeoff of *agr* expression and inhibition can be maintained via genetic mutation within the *agr* locus in a portion of the bacterial population that serves as a “cryptic insurance strategy” [112]. Furthermore, an in vivo study found an infection-type dependence on the benefits of quorum sensing dysfunction. Quorum sensing mutants formed more dense biofilms that facilitated immune evasion and preventing phagocytic attack [120]. On the contrary, *S. aureus* skin-colonizing strains with Agr activity were associated with skin disease, while Agr dysfunction correlated with physiologic skin colonization [121]. These findings are consistent with the ability of *agr* expressing *S. aureus* strains to disseminate from medical devices and distant sites to bone [21,122]. In this sense, temporal and spatial genetic variation may allow *S. aureus* to alter gene expression and adapt to different environments in a way that is evolutionarily sustainable.

Given the important role of the Agr system in coordination of staphylococcal virulence, quenching the ability of *S. aureus* to respond to AIP at quorum may provide a means to ameliorate *S. aureus* disease [123,124]. Diflunisal is one such drug that functions by inhibiting AgrA from binding to DNA as well as preventing phosphorylation of AgrA by AgrC [125]. Diflunisal inhibits PSM production, thereby reducing cell death of osteoblasts exposed to staphylococcal supernatants in vitro [48,125]. When delivered locally or systemically during osteomyelitis, diflunisal decreases cortical bone destruction [48,126]. The small molecule savirin also inhibits *S. aureus* AgrA and prevents host tissue damage in a murine skin infection model [123]. The mechanism of savirin is distinct from diflunisal in that it inhibits the DNA binding capacity of Agr [123]. Other compounds that affect *S. aureus* quorum sensing include the fungal metabolite, ambuic acid, which inhibits AIP production, as well as cyclized dipeptides secreted by *Lactobacillus reuteri*, which are thought to inhibit AgrC/A two-component signaling [127,128]. Furthermore, coagulase-negative skin-resident *Staphylococcus simulans* produce AIP molecules with potent anti-MRSA quorum sensing activity [129]. These studies suggest synthetic and natural anti-Agr compounds might have potential to quench *S. aureus* virulence during infection. However, unwanted side effects of quorum quenching therapies could include increased bacterial binding to host tissues and biofilm formation [18,52,130]. Therefore, further studies are necessary to understand the effects of quorum quenching therapies within infected tissues to determine their effectiveness in altering bone loss observed during *S. aureus* osteomyelitis, a disease classically involving biofilm formation and Agr-mediated toxins.

## 6. Environmental Factors Influence *S. aureus* Agr Regulation Through Crosstalk with Global Regulators

### 6.1. Staphylococcal Respiratory Response AB (SrrAB) Senses and Responds to Changes in Cellular Redox

SrrAB is a *S. aureus* two-component system that becomes activated under conditions of hypoxia and nitrosative stress [12,24,25]. SrrB is a transmembrane histidine kinase that contains domains for dimerization, ATPase activity, and a phosphoacceptor [25]. The redox state of an intramolecular disulfide bond in the ATP-binding domain dictates the autophosphorylation state of SrrB [131]. SrrA is a cytoplasmic response regulator that can bind to DNA and influence transcriptional activity in a SrrB-dependent or -independent mechanism [25]. SrrAB down-regulates RNAIII by binding to the regulatory regions of the P2 and P3 promoters to prevent transcription, thus reducing host cell cytotoxic factor expression in hypoxic conditions [25]. Due to the hypoxic nature of osteomyelitis, SrrAB has a critical role in *S. aureus* stress responses during the process of abscess formation [26]. A *srrAB* mutant strain has a large survival defect during osteomyelitis, and this phenotype is partially rescued by administration of an anti-Ly6G monoclonal antibody, suggesting that SrrAB may regulate *S. aureus* response to stresses induced by neutrophils or abscess formation in vivo. SrrAB also cross-regulates Agr-regulated *psmα* 1–4. Alpha-type PSM production is suppressed through Agr by SrrAB, thus limiting host cell destruction, including osteoblasts [26]. In parallel, activation of SrrAB results in *S. aureus* central metabolism shifts for growth in the absence of respiratory terminal electron receptors and can contribute to development of persistent small colony variants (SCVs) during osteomyelitis [12]. *S. aureus* respiratory changes regulated by SrrAB also result in increased expression of fibronectin-binding protein A (FnBPA), bacterial cell lysis, and release of cytosolic DNA, which enhance biofilm formation [132,133]. Taken together, SrrAB decreases *S. aureus* virulence factor production through cross-regulation of *agr* RNAIII and PSMs but promotes persistence within host tissues by facilitating metabolic adaptations in response to environmental changes in infected host tissues.

### 6.2. Staphylococcal Accessory Regulator A (SarA) Is a Critical Global Regulator that Crosstalks with the Agr System and Contributes to Osteomyelitis Pathogenesis

SarA is a crucial regulator of *S. aureus* biofilm formation that acts by repressing extracellular protease and nuclease production [134,135]. One mechanism by which SarA functions is by binding to and activating transcription of promoter regions, P2 and P3 to upregulate RNAII and RNAIII [136,137,138]. SarA stabilizes regulatory transcripts, *agrA* and *saeS*, as well as a number of virulence factor transcripts, including *hla* (α-hemolysin), *hlb* (β-hemolysin, and *hlgCB* (δ-hemolysin) [23]. However, one of the most notable roles of SarA is its ability to repress extracellular proteases, including aureolysin (Aur), which in turn can degrade PSMs [139,140]. When SarA represses proteases that can degrade PSMs, the overall increase in PSM abundance augments the death of both osteoblasts and osteoclasts. Conversely, inactivation of SarA leads to de-repression of these proteases and a substantial reduction in *S. aureus* virulence [141]. Protease-deficient *S. aureus* is hypervirulent, and mice infected with this mutant in a sepsis model exhibit a drastic decrease in survival, irrespective of the presence of SarA [142]. Therefore, SarA stabilizes Agr-related toxins indirectly by inhibiting degradative proteases in addition to increasing the stability of *saeS* and *agr* transcripts.

### 6.3. The S. aureus Exoprotein Expression (SaeRS) Two-Component System Regulates Proteases that Influence Osteomyelitis-Associated Bone Destruction

SaeRS is a two-component system, comprised of four open reading frames. *SaeS* and *saeR* encode proteins that function as a sensor histidine kinase and a response regulator, and *saeP* and *saeQ* encode proteins that form a complex to bind to SaeS and activate its phosphatase activity [143,144]. Signals known to activate SaeRS include a number of neutrophil-derived antimicrobial defenses, such as hydrogen peroxide associated with phagocytosis, human neutrophil peptides, and calprotectin-bound zinc [145,146]. Conversely, low pH associated with acidic phagosomes has been suggested to inhibit SaeRS in certain *S. aureus* strains [147,148]. Similarly to SarA, SaeRS reduces post-translational degradation of PSMs by downregulating the expression of *aur* [7,139]. This regulation occurs through a SarA-independent mechanism and is dependent on functional SaeS [139]. The effects of SaeRS on PSM degradation were demonstrated in an ex vivo osteoblast culture system, whereby *saeRS* null *S. aureus* exhibited a lack of host cell cytotoxicity. Furthermore, inactivation of *saeRS* decreases PSM-induced cortical bone loss in vivo during osteomyelitis [7].

### 6.4. Metabolite-Responsive Transcription Factors Regulate S. aureus Metabolism and Virulence

CodY is a highly conserved regulatory protein that functions as a transcription factor during stationary phase in Gram-positive pathogens. CodY senses metabolite effectors, including branched-chain amino acids and GTP to regulate central metabolism and virulence gene expression [28,149]. While CodY directly regulates numerous genes involved in amino acid biosynthesis and virulence, it indirectly regulates other virulence genes via repression of the *agr* locus [150]. Several adherence-related genes are activated directly by CodY, including *fnbA* (which encodes the fibronectin-binding protein A) and *spa*. Other virulence genes that are influenced by the Agr system are regulated indirectly by CodY, such as *coa* [149]. Thus, CodY increases Agr-regulated virulence genes associated with *S. aureus* adherence via inhibition of RNAIII transcription as well as directly promoting virulence gene transcription.

A second well-conserved bacterial catabolite-responsive transcription factor is CcpA. CcpA is responsible for controlling glucose metabolism and transcription of select virulence factors in the presence of glucose. Deletion of *ccpA* in *S. aureus* leads to down-regulation of RNAIII, thereby resulting in altered transcription patterns of *hla* and *spa* [151]. The influence of CcpA on virulence factor production provides another crucial link between host metabolic state and *S. aureus* toxin production. This interaction suggests that glucose concentration within the bacterial niche or organ system could drastically alter the virulence profile of *S. aureus*. Glycolysis is a critical pathway that enables *S. aureus* survival in bone, despite the highly glycolytic metabolism of resident bone cells [152,153,154]. The presence of glucose results in preferential use of glycolysis over other pathways such as the tricarboxylic acid (TCA) cycle, pentose phosphate pathway, and gluconeogenesis [154]. Due to the availability of glucose in bone and characterized bacterial glycolytic metabolism during osteomyelitis, CcpA likely influences the ability of *S. aureus* to infect bone, but this remains to be tested [155]. In addition to catabolite responsive regulators, there is also emerging evidence that specific metabolites, such as pyruvate, can regulate Agr activity [156].

## 7. Conclusions

Agr quorum sensing clearly contributes to the pathogenesis of *S. aureus* disease [13,157]. However, the importance of Agr in bone during *S. aureus* osteomyelitis is a subject of ongoing study. The toxins regulated by Agr implicated in osteomyelitis also influence *S. aureus* pathogenesis through similar mechanisms in a number of other tissues, and bone-specific virulence factors have yet to be identified. Virulence regulation in response to Agr quorum sensing is influenced by a number of two-component systems and other regulatory proteins, including those involved in sensing and responding to changes in redox state, immune defenses, and nutrient alterations [12,26,158]. Therefore, in addition to bacterial density, quorum-regulated toxin production is also influenced by the increasingly hypoxic nature of skeletal *S. aureus* abscesses, neutrophilic infiltrate, and the relative abundance of nutrients such as glucose. Select cytotoxins are produced by *S. aureus* in an Agr-dependent manner and influence both bacterial fitness within bone and destruction of bone architecture [7,54,67,71,73,74]. Additionally, immunomodulatory proteins regulated by Agr influence bacterial microcolony formation, adhesion, and defense against immune cells during osteomyelitis [75,88,89,97]. While other cytotoxins, including Leukotoxin ED (LukED) and gamma-hemolysin CB (HlgCB), are positively regulated by *S. aureus* Agr, these leukotoxins have not been studied in the context of *S. aureus* pathogenesis during osteomyelitis [159].

Further research is needed to uncover novel toxins and regulatory mechanisms that facilitate persistent osteomyelitis infections. In particular, while *agr* mutants have been isolated from clinical *S. aureus* osteomyelitis cases, it is unclear when or where the bacteria acquire these genetic changes. The role of Agr in osteomyelitis pathogenesis is likely dependent on the mechanism of disease origin. For example, Agr may be essential for *S. aureus* hematogenous spread to bone, while Agr does not influence bacterial burdens in a post-traumatic osteomyelitis model [7,26]. Additional studies to elucidate the spatial and temporal importance of Agr-regulated genes would both advance the basic science research on *S. aureus* adaptations within a host and inform therapeutic development for quorum quenching therapies. Finally, cross-regulatory proteins, such as CodY and CcpA, have crucial roles in regulating both the expression of central metabolism genes and RNAIII transcription, providing a link between bacterial metabolism and virulence. By expanding our knowledge of metabolic signals in bone during osteomyelitis, we may identify new stimuli that augment *S. aureus* toxin production. Taken together, Agr-regulated genes increase *S. aureus* survival and virulence during infection establishment via toxin production and metabolic adaptations, while inactivation of Agr is associated with persistent infections, highlighting the paradoxical contribution of this regulatory system to osteomyelitis pathogenesis. Further research will reveal additional environmental factors that influence *S. aureus* fitness and toxin production within an infectious nidus during osteomyelitis.

## Figures and Tables

**Table 1:** Critical publications in the field of *Staphylococcus aureus* Agr-regulated toxin production as it relates to osteomyelitis.

Publication	Year	Model	Critical Finding
Gillaspy, A.F. et al. [46]	1995	Rabbit	Agr increases osteomyelitis incidence and severity, but inactivation of *agr* does not inhibit *S. aureus* colonization of bone.
Blevins, J.S. et al. [47]	2003	Mouse	*Agr* mutation decreases osteomyelitis pathology scores, including number of abscesses, physis destruction, and histological inflammation.
Cassat, J.E. et al. [7]	2013	Mouse	Inactivation of *agr* decreases bone destruction, and Agr-regulated α-type PSM production contributes to bone destruction during *S. aureus* infection.
Hendrix, A.S. et al. [48]	2016	Mouse	Pharmacologic blockade of Agr signaling reduces osteomyelitis pathogenesis.
Wilde, A.D. et al. [26]	2015	Mouse	Agr-regulated PSM production is influenced by hypoxia and signaling through SrrAB during osteomyelitis.
Bouras, D. et al. [49]	2018	Human	Clinical pediatric methicillin resistant *S. aureus* (MRSA) isolates primarily contained *agr*-III systems.
Krishna Mannala, G. et al. [50]	2018	Human	A low-virulent clinical *S. aureus* bone isolate harbors frameshift mutations in the *agrC* gene.
Suligoy, C.M. et al. [21]	2018	Mouse	Lack of Agr-dependent factors decrease *S. aureus* virulence during chronic osteomyelitis while permitting adaptation for persistence.
Masters, E.A. et al. [51]	2019	Murine and in vitro	A *S. aureus agr* mutant retains its ability to invade the osteocyte lacuno-canalicular network of cortical bone.
Kwiecinski, J.M. et al. [52]	2019	Human	*S. aureus agr* type from clinical invasive infections correlates with biofilm formation.

## References

[1] Lew D.P., Waldvogel F.A. (2004). Osteomyelitis. Lancet.

[2] Harik N.S., Smeltzer M.S. (2010). Management of acute hematogenous osteomyelitis in children. Expert Rev. Anti Infect. Ther..

[3] Hatzenbuehler J., Pulling T.J. (2011). Diagnosis and management of osteomyelitis. Am. Fam. Physician.

[4] Spagnolo N., Greco F., Rossi A., Ciolli L., Teti A., Posteraro P. (1993). Chronic *staphylococcal osteomyelitis*: A new experimental rat model. Infect. Immun..

[5] Power M.E., Olson M.E., Domingue P.A.G., Costerton J.W. (1990). A rat model of *Staphylococcus aureus* chronic osteomyelitis that provides a suitable system for studying the human infection. J. Med. Microbiol..

[6] Alderson M., Speers D., Emslie K., Nade S. (1986). Acute haematogenous osteomyelitis and septic arthritis—A single disease. A hypothesis based upon the presence of transphyseal blood vessels. J. Bone Jt. Surg. Br..

[7] Cassat J.E., Hammer N.D., Campbell J.P., Benson M.A., Perrien D.S., Mrak L.N., Smeltzer M.S., Torres V.J., Skaar E.P. (2013). A secreted bacterial protease tailors the *Staphylococcus aureus* virulence repertoire to modulate bone remodeling during osteomyelitis. Cell Host Microbe.

[8] Hannan C., Attinger C. (2009). Special considerations in the management of osteomyelitis defects (diabetes, the ischemic or dysvascular bed, and irradiation). Semin. Plast. Surg..

[9] De Mesy Bentley K.L., Trombetta R., Nishitani K., Bello-Irizarry S.N., Ninomiya M., Zhang L., Li C.H., McGrath J.L., Daiss J.L., Awad H.A. (2017). Evidence of *Staphylococcus aureus* deformation, proliferation, and migration in canaliculi of live cortical bone in murine models of osteomyelitis. J. Bone Min. Res..

[10] Lowy F.D. (1998). Medical progress: Staphylococcus aureus infections. N. Engl. J. Med..

[11] Kong C., Neoh H.M., Nathan S. (2016). Targeting staphylococcus aureus toxins: A potential form of anti-virulence therapy. Toxins.

[12] Brandis G., Cao S., Huseby D.L., Hughes D. (2017). Having your cake and eating it-*Staphylococcus aureus* small colony variants can evolve faster growth rate without losing their antibiotic resistance. Microb. Cell.

[13] Jenul C., Horswill A.R. (2018). Regulation of Staphylococcus aureus virulence. Microbiol. Spectr..

[14] Haag A.F., Bagnoli F. (2017). The role of two-component signal transduction systems in *Staphylococcus aureus* virulence regulation. Curr. Top. Microbiol..

[15] Stock A.M., Robinson V.L., Goudreau P.N. (2000). Two-component signal transduction. Annu. Rev. Biochem..

[16] Steiner B.D., Eberly A.R., Hurst M.N., Zhang E.W., Green H.D., Behr S., Jung K., Hadjifrangiskou M. (2018). Evidence of cross-regulation in two closely related pyruvate-sensing systems in uropathogenic Escherichia coli. J. Membr. Biol..

[17] Mike L.A., Choby J.E., Brinkman P.R., Olive L.Q., Dutter B.F., Ivan S.J., Gibbs C.M., Sulikowski G.A., Staugg D.L., Skaar E.P. (2014). Two-component system cross-regulation integrates *Bacillus anthracis* response to heme and cell envelope stress. PLoS Pathog..

[18] Boles B.R., Horswill A.R. (2008). Agr-mediated dispersal of Staphylococcus aureus biofilms. PLoS Pathog..

[19] Sloan T.J., Murray E., Yokoyama M., Massey R.C., Chan W.C., Bonev B.B., Williams P. (2019). Timing is everything: Impact of naturally occurring *Staphylococcus aureus* AgrC cytoplasmic domain adaptive mutations on autoinduction. J. Bacteriol..

[20] Lebeau C., Vandenesch F., Greenland T., Novick R.P., Etienne J. (1994). Coagulase expression in *Staphylococcus aureus* is positively and negatively modulated by an *agr*-dependent mechanism. J. Bacteriol..

[21] Suligoy C.M., Lattar S.M., Noto Llana M., Gonzalez C.D., Alvarez L.P., Robinson D.A., Gomez M.I., Buzzola F.R., Sordelli D.O. (2018). Mutation of *agr* is associated with the adaptation of *Staphylococcus aureus* to the host during chronic osteomyelitis. Front. Cell Infect. Microbiol..

[22] Voyich J.M., Vuong C., De Wald M., Nygaard T.K., Kocianova S., Griffith S., Jones J., Iverson C., Sturdevant D.E., Braughton K.R. (2009). The SaeR/S gene regulatory system is essential for innate immune evasion by *Staphylococcus aureus*. J. Infect. Dis..

[23] Morrison J.M., Anderson K.L., Beenken K.E., Smeltzer M.S., Dunman P.M. (2012). The staphylococcal accessory regulator, SarA, is an RNA-binding protein that modulates the mRNA turnover properties of late-exponential and stationary phase *Staphylococcus aureus* cells. Front. Cell Infect. Microbiol..

[24] Somerville G.A., Proctor R.A. (2009). At the crossroads of bacterial metabolism and virulence factor synthesis in staphylococci. Microbiol. Mol. Biol. Rev..

[25] Pragman A.A., Yarwood J.M., Tripp T.J., Schlievert P.M. (2004). Characterization of virulence factor regulation by SrrAB, a two-component system in *Staphylococcus aureus*. J. Bacteriol..

[26] Wilde A.D., Snyder D.J., Putnam N.E., Valentino M.D., Hammer N.D., Lonergan Z.R., Hinger S.A., Aysanoa E.E., Blanchard C., Dunman P.M. (2015). Bacterial hypoxic responses revealed as critical determinants of the host-pathogen outcome by TnSeq analysis of *Staphylococcus aureus* invasive infection. PLoS Pathog..

[27] Roux A., Todd D.A., Velázquez J.V., Cech N.B., Sonenshein A.L. (2014). CodY-mediated regulation of the *Staphylococcus aureus* Agr system integrates nutritional and population density signals. J. Bacteriol..

[28] Majerczyk C.D., Sadykov M.R., Luong T.T., Lee C., Somerville G.A., Sonenshein A.L. (2008). *Staphylococcus aureus* CodY negatively regulates virulence gene expression. J. Bacteriol..

[29] Ji G., Beavis R.C., Novick R.P. (1995). Cell density control of staphylococcal virulence mediated by an octapeptide pheromone. Proc. Natl. Acad. Sci. USA.

[30] Novick R.R., Projan S.J., Kornblum J., Ross H.F. (1995). The *agr* P2 operon: An autocatalytic sensory transduction system in *Staphylococcus aureus*. Mol. Gen. Genet..

[31] Rajasree K., Fasim A., Gopal B. (2016). Conformational features of the *Staphylococcus aureus* AgrA-promoter interactions rationalize quorum-sensing triggered gene expression. Biochem. Biophys. Rep..

[32] Vandenesch F., Kornblum J., Novick R.P. (1991). A temporal signal, independent of *agr*, is required for *hla* but not *spa* transcription in *Staphylococcus aureus*. J. Bacteriol..

[33] Geisinger E., Adhikari R.P., Jin R., Ross H.F., Novick R.P. (2006). Inhibition of *rot* translation by RNAIII, a key feature of *agr* function. Mol. Microbiol..

[34] Morfeldt E., Taylor D., von Gabain A., Arvidson S. (1995). Activation of alpha-toxin translation in *Staphylococcus aureus* by the trans-encoded antisense RNA, RNAIII. Embo. J..

[35] Queck S.Y., Jameson-Lee M., Villaruz A.E., Bach T.-H.L., Khan B.A., Sturdevant D.E., Ricklefs S.M., Li M., Otto M. (2008). RNAIII-independent target gene control by the Agr quorum-sensing system: Insight into the evolution of virulence regulation in *Staphylococcus aureus*. Mol. Cell.

[36] Arvidson S., Tegmark K. (2001). Regulation of virulence determinants in *Staphylococcus aureus*. Int. J. Med. Microbiol..

[37] Phonimdaen P., O’Reilly M., O’Toole P.W., Foster T.J. (1988). Molecular cloning and expression of the coagulase gene of *Staphylococcus aureus* 8325-4. J. Gen. Microbiol..

[38] Novick R.P., Ross H.F., Projan S.J., Kornblum J., Kreiswirth B., Moghazeh S. (1993). Synthesis of staphylococcal virulence factors is controlled by a regulatory RNA molecule. Embo. J..

[39] Patel A.H., Kornblum J., Kreiswirth B., Novick R., Foster T.J. (1992). Regulation of the protein A-encoding gene in *Staphylococcus aureus*. Gene.

[40] Chabelskaya S., Rie Bordeau V., Felden B. (2014). Dual RNA regulatory control of a *Staphylococcus aureus* virulence factor. Nucleic. Acid.

[41] Liu Y., Mu C., Ying X., Li W., Wu N., Dong J., Gao Y., Shao N., Fan M., Yang G. (2011). RNAIII activates *map* expression by forming an RNA-RNA complex in *Staphylococcus aureus*. Febs. Lett..

[42] Huntzinger E., Boisset S., Saveanu C., Benito Y., Geissmann T., Namane A., Lina K., Etienne J., Ehresmann B., Ehresmann C. (2005). *Staphylococcus aureus* RNAIII and the endoribonuclease III coordinately regulate *spa* gene expression. EMBO J..

[43] Gupta R.K., Luong T.T., Lee C.Y. (2015). RNAIII of the *Staphylococcus aureus agr* system activates global regulator MgrA by stabilizing mRNA. Proc. Natl. Acad. Sci. USA.

[44] Saïd-Salim B., Dunman P.M., McAleese F.M., Macapagal D., Murphy E., McNamara P.J., Arvidson S., Foster T.J., Projan S.J., Kreiswirth B.N. (2003). Global regulation of *Staphylococcus aureus* genes by Rot. J. Bacteriol..

[45] Boisset S., Geissmann T., Huntzinger E., Fechter P., Bendridi N., Possedko M., Chevalier C., Helfer A.C., Benito Y., Jacquier A. (2007). *Staphylococcus aureus* RNAIII coordinately represses the synthesis of virulence factors and the transcription regulator Rot by an antisense mechanism. Genes Dev..

[46] Gillaspy A.F., Hickmon S.G., Skinner R.A., Thomas J.R., Nelson C.L., Smeltzer M.S. (1995). Role of the accessory gene regulator (Agr) in pathogenesis of staphylococcal osteomyelitis. Infect. Immun..

[47] Blevins J.S., Elasri M.O., Allmendinger S.D., Beenken K.E., Skinner R.A., Thomas R.J., Smeltzer M.S. (2003). Role of *sarA* in the Pathogenesis of *Staphylococcus aureus* musculoskeletal infection. Infect. Immun..

[48] Hendrix A.S., Spoonmore T.J., Wilde A.D., Putnam N.E., Hammer N.D., Snyder D.J., Guelcher S.A., Skaar E.P., Cassat J.E. (2016). Repurposing the nonsteroidal anti-inflammatory drug diflunisal as an osteoprotective, antivirulence therapy for *Staphylococcus aureus* osteomyelitis. Antimicrob. Agents Chemother..

[49] Bouras D., Doudoulakakis A., Tsolia M., Vaki I., Giormezis N., Petropoulou N., Lebessi E., Gennimata V., Tsakris A., Spiliopoulou I. (2018). *Staphylococcus aureus* osteoarticular infections in children: An 8-year review of molecular microbiology, antibiotic resistance and clinical characteristics. J. Med. Microbiol..

[50] Krishna Mannala G., Koettnitz J., Mohamed W., Sommer U., Lips K.S., Sproer C., Bunk B., Overmann J., Hain T., Heiss C. (2018). Whole-genome comparison of high and low virulent *Staphylococcus aureus* isolates inducing implant-associated bone infections. Int. J. Med. Microbiol..

[51] Masters E.A., Salminen A.T., Begolo S., Luke E.N., Barrett S.C., Overby C.T., Gill A.L., de Mesy Bentley K.L., Awad H.A., Gill S.R. (2019). An in vitro platform for elucidating the molecular genetics of *S. aureus* invasion of the osteocyte lacuno-canalicular network during chronic osteomyelitis. Nanomedicine.

[52] Kwiecinski J.M., Jacobsson G., Horswill A.R., Josefsson E., Jin T. (2019). Biofilm formation by *Staphylococcus aureus* clinical isolates correlates with the infection type. Infect. Dis..

[53] Peschel A., Otto M. (2013). Phenol-soluble modulins and staphylococcal infection. Nat. Rev. Microbiol..

[54] Chatterjee S.S., Joo H.S., Duong A.C., Dieringer T.D., Tan V.Y., Song T., Fischer E.R., Cheung G.Y.C., Li M., Otto M. (2013). Essential *Staphylococcus aureus* toxin export system. Nat. Med..

[55] Rasigade J.-P., Trouillet-Assant S., Ferry T., Diep B.A., Sapin A., Lhoste Y., Ranfaing J., Badiou C., Bento Y., Bes M. (2013). PSMs of hypervirulent *Staphylococcus aureus* act as intracellular toxins that kill infected osteoblasts. PLoS ONE.

[56] Kretschmer D., Gleske A.-K., Rautenberg M., Wang R., Koberle M., Bohn E., Schoneberg T., Rabiet M.-J., Boulay F., Klebanoff S.J. (2010). Human formyl peptide receptor 2 (FPR2/ALX) senses highly pathogenic *Staphylococcus aureus*. Cell Host Microbe..

[57] Surewaard B.G.J., De Haas C.J.C., Vervoort F., Rigby K.M., Deleo F.R., Otto M., Van Stijp J.A.G., Nijland R. (2013). Staphylococcal alpha-phenol soluble modulins contribute to neutrophil lysis after phagocytosis. Cell Microbiol..

[58] Brandt S.L., Putnam N.E., Cassat J.E., Serezani H. (2019). Innate immunity to *Staphylococcus aureus*: Evolving paradigms in soft tissue and invasive infection. J. Immunol..

[59] Füssle R., Bhakdi S., Sziegoleit A., Tranum-Jensen J., Kranz T., Wellensiek H.-J. (1981). On the mechanism of membrane damage by *Staphylococcus aureus* a-Toxin. J. Cell Biol..

[60] Ward R.J., Leonard K. (1992). The *Staphylococcus aureus* α-toxin channel complex and the effect of Ca^2+^ ions on its interaction with lipid layers. J. Struct. Biol..

[61] Tobkes N., Wallace B.A., Bayley H. (1985). Secondary structure and assembly mechanism of an oligomeric channel protein. Biochemistry.

[62] Wilke G.A., Wardenburg J.B. (2010). Role of a disintegrin and metalloprotease 10 in *Staphylococcus aureus* α-hemolysin-mediated cellular injury. Proc. Natl. Acad. Sci. USA.

[63] Berube B.J., Wardenburg J.B. (2013). *Staphylococcus aureus* α-toxin: Nearly a century of intrigue. Toxins.

[64] Ma J., Gulbins E., Edwards M.J., Caldwell C.C., Fraunholz M., Becker K.A. (2017). *Staphylococcus aureus* α-toxin induces inflammatory cytokines via lysosomal acid sphingomyelinase and ceramides. Cell. Physiol. Biochem..

[65] Craven R.R., Gao X., Allen I.C., Gris D., Bubeck Wardenburg J., McElvania-TeKippe E., Ting J.P., Duncan J.A. (2009). *Staphylococcus aureus* α-hemolysin activates the NLRP3-inflammasome in human and mouse monocytic cells. PLoS ONE.

[66] Nilsson I.M., Hartford O., Foster T., Tarkowski A. (1999). Alpha-toxin and gamma-toxin jointly promote *Staphylococcus aureus* virulence in murine septic arthritis. Infect. Immun..

[67] Smith I.D.M., Milto K.M., Doherty C.J., Amyes S.G.B., Simpson A.H.R.W., Hall A.C. (2018). A potential key role for alpha-haemolysin of *Staphylococcus aureus* in mediating chondrocyte death in septic arthritis. Bone Jt. Res..

[68] Yoong P., Torres V.J. (2013). The effects of *Staphylococcus aureus* leukotoxins on the host: Cell lysis and beyond. Curr. Opin. Microbiol..

[69] Colin D.A., Mazurier I., Sire S., Finck-Barbancon V. (1994). Interaction of the two components of leukocidin from *Staphylococcus aureus* with human polymorphonuclear leukocyte membranes: Sequential binding and subsequent activation. Infect. Immun..

[70] Menestrina G., Dalla Serra M., Comai M., Coraiola M., Viero G., Werner S., Colin D.A., Monteil H., Prevost G. (2003). Ion channels and bacterial infection: The case of β-barrel pore-forming protein toxins of *Staphylococcus aureus*. FEBS Lett..

[71] Cré Mieux A.-C., Dumitrescu O., Lina G., Vallee C., Cote J.-F., Muffat-Joly M., Lilin T., Etienne J., Vandenesch F., Saleh-Mghir A. (2019). Panton-Valentine leukocidin enhances the severity of community-associated methicillin-resistant *Staphylococcus aureus* rabbit osteomyelitis. PLoS ONE.

[72] Holzinger D., Gieldon L., Mysore V., Nippe N., Taxman D.J., Duncan J., Broglie P.M., Marketon K., Austermann J., Vogl T. (2012). *Staphylococcus aureus* Panton-Valentine leukocidin induces an inflammatory response in human phagocytes via the NLRP3 inflammasome. J. Leukoc. Biol..

[73] Jiang B., Wang Y., Feng Z., Xu L., Zhao S., Gong Y., Zhang C., Luo X., Li S., Rao X. (2017). Panton-Valentine leucocidin (PVL) as a potential indicator for prevalence, duration, and severity of *Staphylococcus aureus* osteomyelitis. Front. Microbiol..

[74] Bocchini C.E., Hulten K.G., Mason E.O., Gonzalez B.E., Hammerman W.A., Kaplan S.L. (2006). Panton-Valentine leukocidin genes are associated with enhanced inflammatory response and local disease in acute hematogenous *Staphylococcus aureus* osteomyelitis in children. Pediatrics.

[75] Jin T., Zhu Y., Li J., Shi J., He X.Q., Ding J., Xu Y.Q. (2013). Staphylococcal protein A, Panton-Valentine leukocidin and coagulase aggravate the bone loss and bone destruction in osteomyelitis. Cell Physiol. Biochem..

[76] Dohin B., Gillet Y., Kohler R., Lina G., Vandenesch F., Vanhems P., Floret D., Etienne J. (2007). Pediatric bone and joint infections caused by Panton-Valentine leukocidin-positive *Staphylococcus aureus*. Pediatr. Infect. Dis. J..

[77] Wardenburg J.B., Palazzolo-Ballance A.M., Otto M., Schneewind O., DeLeo F.R. (2008). Panton-Valentine leukocidin is not a virulence determinant in murine models of community-associated methicillin-resistant *Staphylococcus aureus* disease. J. Infect. Dis..

[78] Tromp A.T., Van Gent M., Abrial P., Martin A., Jansen J.P., De Haas C.J.C., Van Kessel K.P.M., Bardoel B.W., Kruse E., Bourdonnay E. (2018). Human CD45 is an f-component-specific receptor for the staphylococcal toxin Panton-Valentine leukocidin. Nat. Microbiol..

[79] Löffler B., Hussain M., Grundmeier M., Bruck M., Holzinger D., Varga G., Roth J., Kahl B.C., Proctor R.A., Peter G. (2010). *Staphylococcus aureus* Panton-Valentine leukocidin is a very potent cytotoxic factor for human neutrophils. PLoS Pathog..

[80] Dossett J.H., Kronvall G., Williams R.C., Quie P.G. (1969). RNAIII of the *Staphylococcus aureus* agr system activates global regulator MgrA by stabilizing mRNA. J. Immunol..

[81] Kobayashi S.D., DeLeo F.R. (2013). *Staphylococcus aureus* protein A promotes immune suppression. MBio.

[82] Goodyear C.S., Silverman G.J. (2003). Death by a B cell superantigen: In vivo VH-targeted apoptotic supraclonal B cell deletion by a staphylococcal toxin. J. Exp. Med..

[83] Keener A.B., Thurlow L.T., Kang S., Spidale N.A., Clarke S.H., Cunnion K.M., Tisch R., Richardson A.R., Vilen B.J. (2017). *Staphylococcus aureus* protein A disrupts immunity mediated by long-lived plasma cells. J. Immunol..

[84] Claro T., Widaa A., O’Seaghdha M., Miajlovic H., Foster T.J., O’Brien F.J., Kerrigan S.W. (2011). *Staphylococcus aureus* Protein A binds to osteoblasts and triggers signals that weaken bone in osteomyelitis. PLoS ONE.

[85] Claro T., Widaa A., McDonnell C., Foster T.J., O’Brien F.J., Kerrigan S.W. (2013). *Staphylococcus aureus* protein A binding to osteoblast tumour necrosis factor receptor 1 results in activation of nuclear factor kappa B and release of interleukin-6 in bone infection. Microbiology.

[86] Widaa A., Claro T., Foster T.J., O’Brien F.J., Kerrigan S.W. (2012). *Staphylococcus aureus* protein A plays a critical role in mediating bone destruction and bone loss in osteomyelitis. PLoS ONE.

[87] Mendoza Bertelli A., Delpino M.V., Lattar S., Giai C., Noto Llana M., Sanjuan N., Cassat J.E., Sordelli D., Gomez M. (2016). *Staphylococcus aureus* protein A enhances osteoclastogenesis via TNFR1 and EGFR signaling. Biochim. Biophys. Acta.

[88] Chavakis T., Hussain M., Kanse S.M., Peters G., Bretzel R.G., Flock J.-I., Herrmann M., Preissner K.T. (2002). *Staphylococcus aureus* extracellular adherence protein serves as anti-inflammatory factor by inhibiting the recruitment of host leukocytes. Nat. Med..

[89] Haggar A., Ehrnfelt C., Holgersson J., Flock J.-I. (2004). The extracellular adherence protein from *Staphylococcus aureus* inhibits neutrophil binding to endothelial cells. Infect. Immun..

[90] Lee L.Y., Miyamoto Y.J., McIntyre B.W., Hook M., McCrea K.W., McDevill D., Brown E.L. (2002). The *Staphylococcus aureus* Map protein is an immunomodulator that interferes with T cell-mediated responses. J. Clin. Investig..

[91] Wolz C., McDevitt D., Foster T.J., Cheung A.L. (1996). Influence of *agr* on fibrinogen binding in *Staphylococcus aureus* Newman. Infect. Immun..

[92] Xue T., You Y., Shang F., Sun B. (2012). Rot and Agr system modulate fibrinogen-binding ability mainly by regulating *clfB* expression in *Staphylococcus aureus* NCTC8325. Med. Microbiol. Immunol..

[93] Dunman P.M., Murphy E., Haney S., Palacios D., Tucker-Kellogg G., Wu S., Brown E.L., Zagursky R.J., Shlaes D., Projan S.J. (2001). Transcription profiling-based identification of *Staphylococcus aureus* genes regulated by the *agr* and/or *sarA* loci. J. Bacteriol..

[94] Cunningham R., Cockayne A., Humphreys H. (1996). Clinical and molecular aspects of the pathogenesis of *Staphylococcus aureus* bone and joint infections. J. Med. Microbiol..

[95] Panizzi P., Friedrich R., Fuentes-Prior P., Richter K., Bock P.E., Bode W. (2006). Fibrinogen substrate recognition by staphylocoagulase (pro)thrombin complexes. J. Biol. Chem..

[96] Cheng A.G., McAdow M., Kim H.K., Bae T., Missiakas D.M., Schneewind O. (2010). Contribution of coagulases towards *Staphylococcus aureus* disease and protective immunity. PLoS Pathog..

[97] Guggenberger C., Wolz C., Morrissey J.A., Heesemann J. (2012). Two distinct coagulase-dependent barriers protect *Staphylococcus aureus* from neutrophils in a three dimensional in vitro infection model. PLoS Pathog..

[98] Thomas S., Liu W., Arora S., Ganesh V., Ko Y.-P., Hook M. (2019). The complex fibrinogen interactions of the *Staphylococcus aureus* coagulases. Front. Cell Infect. Microbiol..

[99] Dufour P., Jarraud S., Vandenesch F., Greenland T., Novick R.P., Bes M., Etienee J., Lina G. (2002). High genetic variability of the *agr* locus in *Staphylococcus* species. J. Bacteriol..

[100] Jarraud S., Lyon G.J., Figueiredo A.M., Lina G., Vandenesch F., Etienee J., Muir T.W., Novick R.P. (2000). Exfoliatin-producing strains define a fourth *agr* specificity group in *Staphylococcus aureus*. J. Bacteriol..

[101] Guangyong J., Beavis R., Novick R. (1997). Bacterial interference caused by autoinducing peptide variants. Science.

[102] Zhang L., Ji G. (2004). Identification of a staphylococcal AgrB segment(s) responsible for group-specific processing of AgrD by gene swapping. J. Bacteriol..

[103] Nair N., Biswas R., Götz F., Biswas L. (2014). Impact of *Staphylococcus aureus* on pathogenesis in polymicrobial infections. Infect. Immune..

[104] Paharik A.E., Parlet C.P., Chung N., Todd D.A., Rodriguez L.I., Van Dyke M.J., Cech N.B., Horswill A.R. (2017). Coagulase-negative Staphylococcal strain prevents *Staphylococcus aureus* colonization and skin infection by blocking quorum sensing. Cell Host Microbe.

[105] Souza Jorge L., Silva Fucuta P., Oliveira M.G.L., Arruda Nakazone M., Arruda de Matos J., Gomes Chueire A., Costa Salles M.J. (2018). Outcomes and risk factors for polymicrobial posttraumatic osteomyelitis. J. Bone Joint. Infect..

[106] Matias C., Serrano I., Van-Harten S., Mottola C., Mendes J.J., Tavares L., Oliveira M. (2018). Polymicrobial interactions influence the *agr* copy number in *Staphylococcus aureus* isolates from diabetic foot ulcers. Antonie Van Leeuwenhoek.

[107] Todd O.A., Peters B.M. (2019). *Candida albicans* and *Staphylococcus aureus* pathogenicity and polymicrobial interactions: Lessons beyond Koch’s postulates. J. Fungi..

[108] Shopsin B., Drlica-Wagner A., Mathema B., Adhikari R.P., Kreiswirth B.N., Novick R.P. (2008). Prevalence of *agr* dysfunction among colonizing *Staphylococcus aureus* strains. J. Infect. Dis..

[109] Traber K.E., Lee E., Benson S., Corrigan R., Cantera M., Shopsin B., Novick R.P. (2008). *Agr* function in clinical *Staphylococcus aureus* isolates. Microbiology.

[110] Schweizer M.L., Furuno J.P., Sakoulas G., Johnson J.K., Harris A.D., Shardell M.D., McGregor J.C., Thom K.A., Perencevich E.N. (2011). Increased mortality with accessory gene regulator (*agr*) dysfunction in *Staphylococcus aureus* among bacteremic patients. Antimicrob. Agents Chemother..

[111] Altman D.R., Sullivan M.J., Chacko K.I., Balasubramanian D., Pak T.R., Sause W.E., Kumar K., Sebra R., Deikus G., Attie O. (2018). Genome plasticity of *agr*-defective *Staphylococcus aureus* during clinical infection. Infect. Immun..

[112] Gor V., Takemura A.J., Nishitani M., Higashide M., Medrano Romero V., Ohniwa R.L., Morikawa K. (2019). Finding of Agr phase variants in *Staphylococcus aureus*. MBio.

[113] Buckling A., Neilson J., Lindsay J., ffrench-Constant R., Enright M., Day N., Massey R.C. (2005). Clonal distribution and phase-variable expression of a major histocompatibility complex analogue protein in *Staphylococcus aureus*. J. Bacteriol..

[114] Traber K., Novick R. (2006). A slipped-mispairing mutation in AgrA of laboratory strains and clinical isolates results in delayed activation of *agr* and failure to translate δ- and α-haemolysins. Mol. Microbiol..

[115] Cheng A.G., DeDent A.C., Schneewind O., Missiakas D. (2011). A play in four acts: *Staphylococcus aureus* abscess formation. Trends Microbiol..

[116] Carnes E.C., Lopez D.M., Donegan N.P., Donegan N.P., Cheung A., Gresham H., Timmins G.S., Brinker C.J. (2010). Confinement-induced quorum sensing of individual *Staphylococcus aureus* bacteria. Nat. Chem. Biol..

[117] Kolar S.L., Antonio Ibarra J., Rivera F.E., Mootz J.M., Davenport J.E., Stevens S.M., Horswill A.R., Shaw L.N. (2013). Extracellular proteases are key mediators of *Staphylococcus aureus* virulence via the global modulation of virulence-determinant stability. Microbiologyopen.

[118] Fowler V.G., Sakoulas G., Mcintyre L.M., Meka V.G., Arbeit R.D., Cabell C.H., Stryjewski M.E., Eliopoulos G.M., Reller B., Corey R. (2004). Persistent bacteremia due to methicillin-resistant *Staphylococcus aureus* infection is associated with *agr* dysfunction and low-level in vitro resistance to thrombin-induced platelet microbicidal protein. JID.

[119] Paulander W., Varming A.N., Bæk K.T., Haaber J., Frees D., Ingmer H. (2012). Antibiotic-mediated selection of quorum-sensing-negative *Staphylococcus aureus*. Mbio.

[120] He L., Le K.Y., Khan B.A., Nguyen T.H., Hung R.L., Bae J.S., Kabat J., Zheng Y., Cheung G.Y.C., Li M. (2019). Resistance to leukocytes ties benefits of quorum sensing dysfunctionality to biofilm infection. Nat. Microbiol..

[121] Nakamura Y., Takahashi H., Takaya A., Inoue Y., Katayama Y., Kusuya Y., Shoji T., Takada S., Nakagawa S., Oguma R. (2020). *Staphylococcus* Agr virulence is critical for epidermal colonization and associates with atopic dermatitis development. Sci. Transl. Med..

[122] Periasamy S., Joo H.-S., Duong A.C., Bach T.-H.L., Tan V.Y., Chatterjee S.S., Cheung G.Y.C., Otto M. (2012). How *Staphylococcus aureus* biofilms develop their characteristic structure. Proc. Natl. Acad. Sci. USA.

[123] Sully E.K., Malachowa N., Elmore B.O., Alexander S.M., Femling J.K., Gray B.M., DeLeo F.R., Otto M., Cheung A.L., Edwards B.S. (2014). Selective chemical inhibition of *agr* quorum sensing in *Staphylococcus aureus* promotes host defense with minimal impact on resistance. PLoS Pathog..

[124] Gray B., Hall P., Gresham H. (2013). Targeting *agr*- and *agr*-like quorum sensing systems for development of common therapeutics to treat multiple gram-positive bacterial infections. Sensors.

[125] Khodaverdian V., Pesho M., Truitt B., Bollinger L., Patel P., Nithianantham S., Yu G., Delaney E., Jankowski E., Shoham M. (2013). Discovery of antivirulence agents against methicillin-resistant *Staphylococcus aureus*. Antimicrob. Agents Chemother..

[126] Spoonmore T.J., Ford C.A., Curry J.M., Guelcher S.A., Cassat J.E. (2020). Concurrent local delivery of diflunisal limits bone destruction but fails to improve systemic vancomycin efficacy during *Staphylococcus aureus* osteomyelitis. Antimicrob. Agents Chemother..

[127] Nakayama J., Uemura Y., Nishiguchi K., Yoshimura N., Igarashi Y., Sonomoto K. (2009). Ambuic acid inhibits the biosynthesis of cyclic peptide quormones in gram-positive bacteria. Antimicrob. Agents Chemother..

[128] Li J., Wang W., Xu S.X., Magarvey N.A., McCormick J.K. (2011). *Lactobacillus reuteri*-produced cyclic dipeptides quench Agr-mediated expression of toxic shock syndrome toxin-1 in staphylococci. Proc. Natl. Acad. Sci. USA.

[129] Brown M.M., Kwiecinski J.M., Cruz L.M., Shahbandi A., Todd D.A., Cech N.B., Horswill A.R. (2020). Novel peptide from commensal *Staphylococcus simulans* blocks methicillin-resistant *Staphylococcus aureus* quorum sensing and protects host skin from damage. Antimicrob. Agents Chemother..

[130] Valour F., Rasigade J.P., Trouillet-Assant S., Gagnaire J., Bouaziz A., Karsenty J., Lacour C., Bes M., Lustig S., Benet T. (2015). Delta-toxin production deficiency in *Staphylococcus aureus*: A diagnostic marker of bone and joint infection chronicity linked with osteoblast invasion and biofilm formation. Clin. Microbiol. Infect..

[131] Tiwari N., López-Redondo M., Miguel-Romero L., Kulhankova K., Cahill M.P., Tran P.M., Kinney K.J., Kilgore S.H., Al-Tameemi H., Herfst C.A. (2020). The SrrAB two-component system regulates *Staphylococcus aureus* pathogenicity through redox sensitive cysteines. Proc. Natl. Acad. Sci. USA.

[132] Mashruwala A.A., van de Guchte A., Boyd J.M. (2017). Impaired respiration elicits SrrAB-dependent programmed cell lysis and biofilm formation in *Staphylococcus aureus*. Elife.

[133] Mashruwala A.A., Gries C.M., Scherr T.D., Kielian T., Boyd J.M. (2017). SaeRS is responsive to cellular respiratory status and regulates fermentative biofilm formation in *Staphylococcus aureus*. Infect. Immun..

[134] Beenken K.E., Mrak L.N., Griffin L.M., Zielinska A.K., Shaw L.N., Rice K.C., Horswill A.R., Bayles K.W., Smeltzer M.S. (2010). Epistatic relationships between *sarA* and *agr* in *Staphylococcus aureus* biofilm formation. PLoS ONE.

[135] Cheung A.L., Koomey J.M., Butler C.A., Projan S.J., Fischetti V.A. (1992). Regulation of exoprotein expression in *Staphylococcus aureus* by a locus (*sar*) distinct from *agr*. Proc. Natl. Acad. Sci. USA.

[136] Cheung A.L., Projan S.J. (1994). Cloning and sequencing of *sarA* of *Staphylococcus aureus*, a gene required for the expression of *agr*. J. Bacteriol..

[137] Chien Y.T., Manna A.C., Cheung A.L. (1998). SarA level is a determinant of *agr* activation in *Staphylococcus aureus*. Mol. Microbiol..

[138] Reyes D., Andrey D.O., Monod A., Kelley W.L., Zhang G., Cheung A.L. (2011). Coordinated regulation by AgrA, SarA, and SarR to control *agr* expression in *Staphylococcus aureus*. J. Bacteriol..

[139] Zielinska A.K., Beenken K.E., Joo H.-S., Mrak L.N., Griffin L.M., Luong T.T., Lee C.Y., Otto M., Shaw L.N., Smeltzer M.S. (2011). Defining the strain-dependent impact of the staphylococcal accessory regulator (*sarA*) on the alpha-toxin phenotype of *Staphylococcus aureus*. J. Bacteriol..

[140] Lindsay J.A., Foster S.J. (1999). Interactive regulatory pathways control virulence determinant production and stability in response to environmental conditions in *Staphylococcus aureus*. Mol. Gen. Genet..

[141] Loughran A.J., Gaddy D., Beenken K.E., Meeker D.G., Morell R., Zhao H., Byrum S.D., Tackett A.J., Cassat J.E., Smeltzer M.S. (2016). Impact of *sarA* and phenol-soluble modulins on the pathogenesis of osteomyelitis in diverse clinical isolates of *Staphylococcus aureus*. Infect. Immun..

[142] Zielinska A.K., Beenken K.E., Mrak L.N., Spencer H.J., Post G.R., Skinner R.A., Tackett A.J., Horswill A.R., Smeltzer M.S. (2012). SarA-mediated repression of protease production plays a key role in the pathogenesis of *Staphylococcus aureus* USA300 isolates. Mol. Microbiol..

[143] Sun F., Li C., Jeong D., Sohn C., He C., Bae T. (2010). In the *Staphylococcus aureus* two-component system *sae*, the response regulator SaeR Binds to a direct repeat sequence and DNA binding requires phosphorylation by the sensor kinase SaeS. J. Bacteriol..

[144] Jeong D.W., Cho H., Jones M.B., Shatzkes K., Sun F., Ji Q., Liu Q., Peterson S.N., He C., Bae T. (2012). The auxiliary protein complex SaePQ activates the phosphatase activity of sensor kinase SaeS in the SaeRS two-component system of *Staphylococcus aureus*. Mol. Microbiol..

[145] Geiger T., Goerke C., Mainiero M., Kraus D., Wolz C. (2008). The virulence regulator Sae of *Staphylococcus aureus*: Promoter activities and response to phagocytosis-related signals. J. Bacteriol..

[146] Cho H., Jeong D.W., Liu Q., Yeo W.-S., Vogl T., Skaar E.P., Chazen W.J., Bae T. (2015). Calprotectin increases the activity of the SaeRS two component system and murine mortality during *Staphylococcus aureus* infections. PLoS Pathog..

[147] Weinrick B., Dunman P.M., McAleese F., Murphy E., Projan S.J., Fang Y., Novick R.P. (2004). Effect of mild acid on gene expression in *Staphylococcus aureus*. J. Bacteriol..

[148] Geiger T., Francois P., Liebeke M., Fraunholz M., Goerke C., Krismer B., Schrenzel J., Lalk M., Wolz C. (2012). The stringent response of *Staphylococcus aureus* and its impact on survival after phagocytosis through the induction of intracellular PSMs expression. PLoS Path..

[149] Pohl K., Francois P., Stenz L., Schlink F., Geiger T., Herbert C.G., Schrenzel J., Wolz C. (2009). CodY in *Staphylococcus aureus*: A regulatory link between metabolism and virulence gene expression. J. Bacteriol..

[150] Majerczyk C.D., Dunman P.M., Luong T.T., Lee C.Y., Sadykov M.R., Somerville G.A., Bodi K., Sonenshein A.L. (2010). Direct targets of CodY in *Staphylococcus aureus*. J. Bacteriol..

[151] Seidl K., Stucki M., Rueg M., Goerke C., Wolz C., Harris L., Berger-Bachi B., Bischoff M. (2006). *Staphylococcus aureus* CcpA affects virulence determinant production and antibiotic resistance. Antimicrob. Agents Chemother..

[152] Esen E., Long F. (2014). Aerobic glycolysis in osteoblasts. Curr. Osteoporos. Rep..

[153] Kim J.-M., Jeong D., Kang H.K., Jung S.Y., Kang S.S., Min B.-M. (2007). Osteoclast precursors display dynamic metabolic shifts toward accelerated glucose metabolism at an early stage of RANKL-stimulated osteoclast differentiation. Cell Physiol. Biochem..

[154] Potter A.D., Butrico C.E., Ford C.A., Curry J.M., Trenary I.A., Tummarakota S.S., Hendrix A.S., Young J.D., Cassat J.E. (2020). Host nutrient milieu drives an essential role for aspartate biosynthesis during invasive *Staphylococcus aureus* infection. Proc. Natl. Acad. Sci. USA.

[155] Bischoff M., Wonnenberg B., Nippe N., Nyffenegger-Jann N.J., Voss M., Beisswenger C., Sunderkotter C., Molle V., Dinh Q.T., Lammert F. (2017). CcpA affects infectivity of *Staphylococcus aureus* in a hyperglycemic environment. Front. Cell Infect. Microbiol..

[156] Harper L., Balasubramanian D., Ohneck E.A., Sause W.E., Chapman J., Mejia-Sosa B., Lhakhang T., Heguy A., Tsirigos A., Ueberheide B. (2018). *Staphylococcus aureus* responds to the central metabolite pyruvate to regulate virulence. MBio.

[157] Ky L., Otto M. (2015). Quorum-sensing regulation in staphylococci-an overview. Front. Microbiol..

[158] Mlynek K.D., Sause W.E., Moormeier D.E., Sadykov M.R., Hill K.R., Torres V.J., Bayles K.W., Brinsmade S.R. (2018). Nutritional regulation of the Sae two-component system by CodY in *Staphylococcus aureus*. J. Bacteriol..

[159] Bronner S., Stoessel P., Gravet A., Monteil H., Prevost G. (2000). Variable expressions of *Staphylococcus aureus* bicomponent leucotoxins semiquantified by competitive reverse transcription-PCR. Appl. Environ. Microbiol..

